# Dolichoectasia and Its Diagnostic Criteria: A Case Report and Literature Review

**DOI:** 10.7759/cureus.12516

**Published:** 2021-01-06

**Authors:** Jacques M Conradie, Embrensia G Bonnet

**Affiliations:** 1 Neurosurgery, Robert Mangaliso Sobukwe Hospital, Kimberley, ZAF

**Keywords:** dolichoectasia, aneurysm, vasculopathy

## Abstract

Dolichoectasia (DE) is a rare disorder of cerebral vasculature and involves dilation and elongation of the blood vessels. It is mostly reported in the vertebrobasilar circulation, but it can occur in the anterior circulation. This report describes a case involving both anterior and posterior vessel dilation with the suspicion of DE. Here the vessels were enlarged - but not grossly - as in some cases where the diagnosis is obvious. Thus a closer look had to be taken. We refer to multiple studies that attempt to provide some guideline for diagnosis assisting us with our assessment. This illustrates the importance of objective evaluation to prevent missing important pathologies that can change treatment and prognosis if identified.

## Introduction

The prevalence of dolichoectasia has been reported to be 0.05-0.06% with preferential involvement of the vertebrobasilar circulation [1] in contrast with the relatively rare involvement of the anterior circulation [2]. Diffuse intracranial dolichoectasia (anterior and posterior) is extremely scarce and considered to represent a distinct vascular phenotype from isolated vertebrobasilar dolichoectasia [3]. The diagnosis is made radiologically with CT angiography in most cases, but magnetic resonance angiography has been described in some [4]. Different radiological criteria have been suggested to better define the radiological diagnosis. Arterial hypertension and atherosclerosis have been implicated as possible aetiologies, but recently a genetic predisposition has also been implicated [5].

## Case presentation

A 58-year-old female presented to the emergency department with a history of generalized tonic-clonic seizure at home followed by persistent confusion since the incident. No history of recent trauma was noted. She was known with hypertension and chronic obstructive pulmonary disease (COPD) attributed to a smoking history. She was not receiving any anti-hypertensive treatment and was only taking a steroid inhaler at home for the COPD. Further, she was not known with any other co-morbidites, including Human Immunodeficiency Virus (HIV). 

In the emergency department she had another generalized tonic-clonic seizure that was aborted with 4mg of midazolam and was taken for an emergency CT brain. Both a contrast and non-contrast CT was performed, and angiography was added.

**Figure 1:**
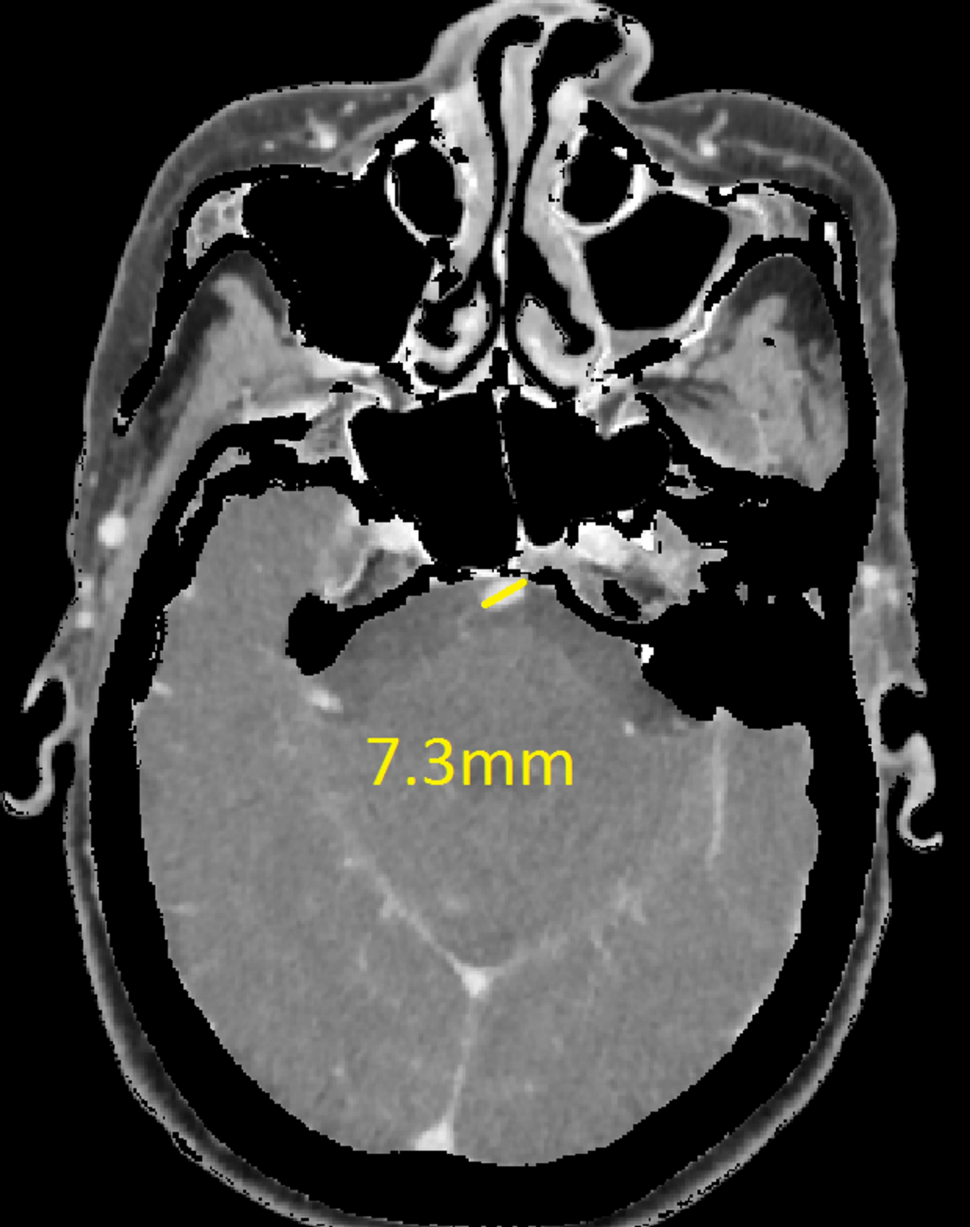
Basilar artery diameter at level of mid pons

**Figure 2:**
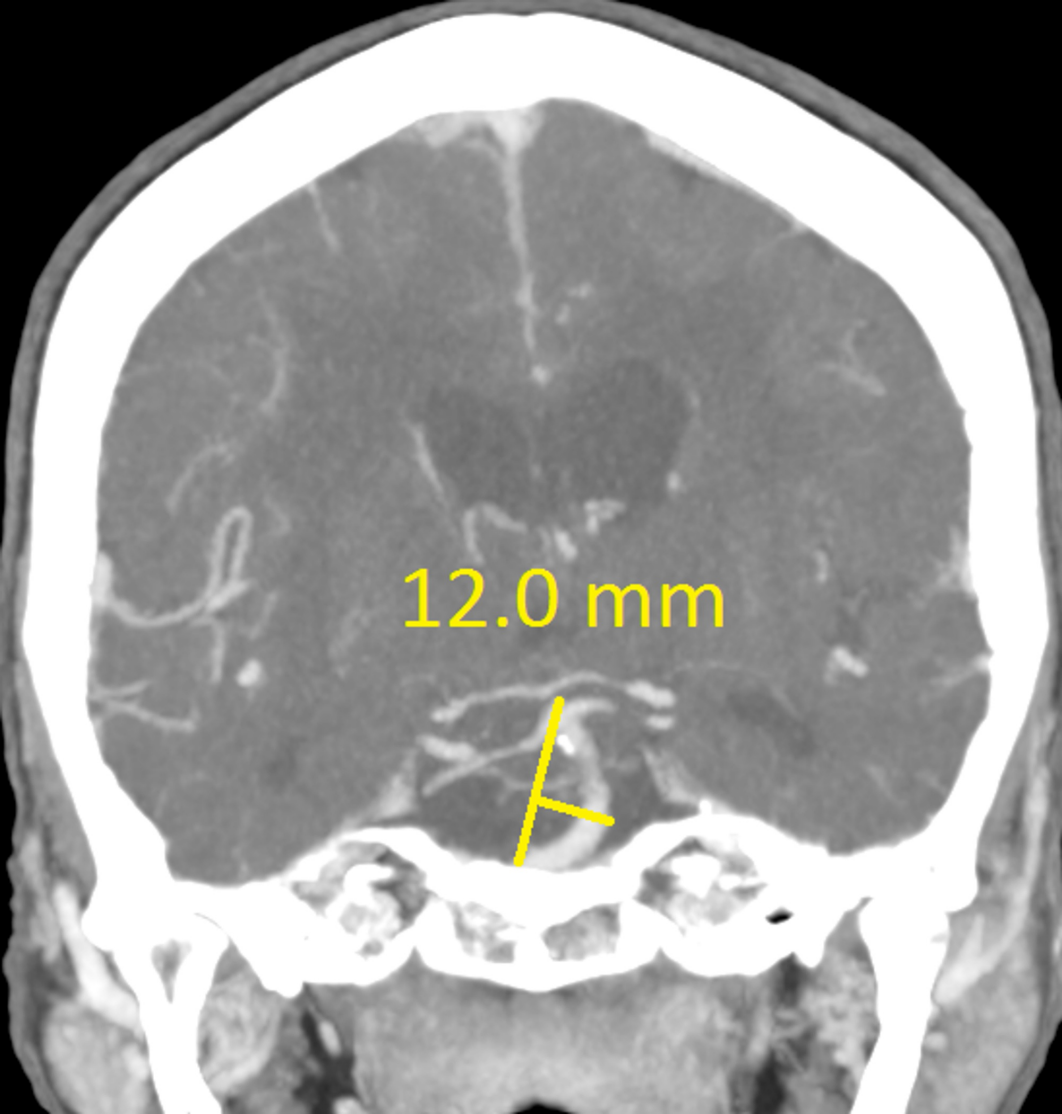
Basilar artery deviation from line connecting basilar artery origin and its bifurcation

**Figure 3:**
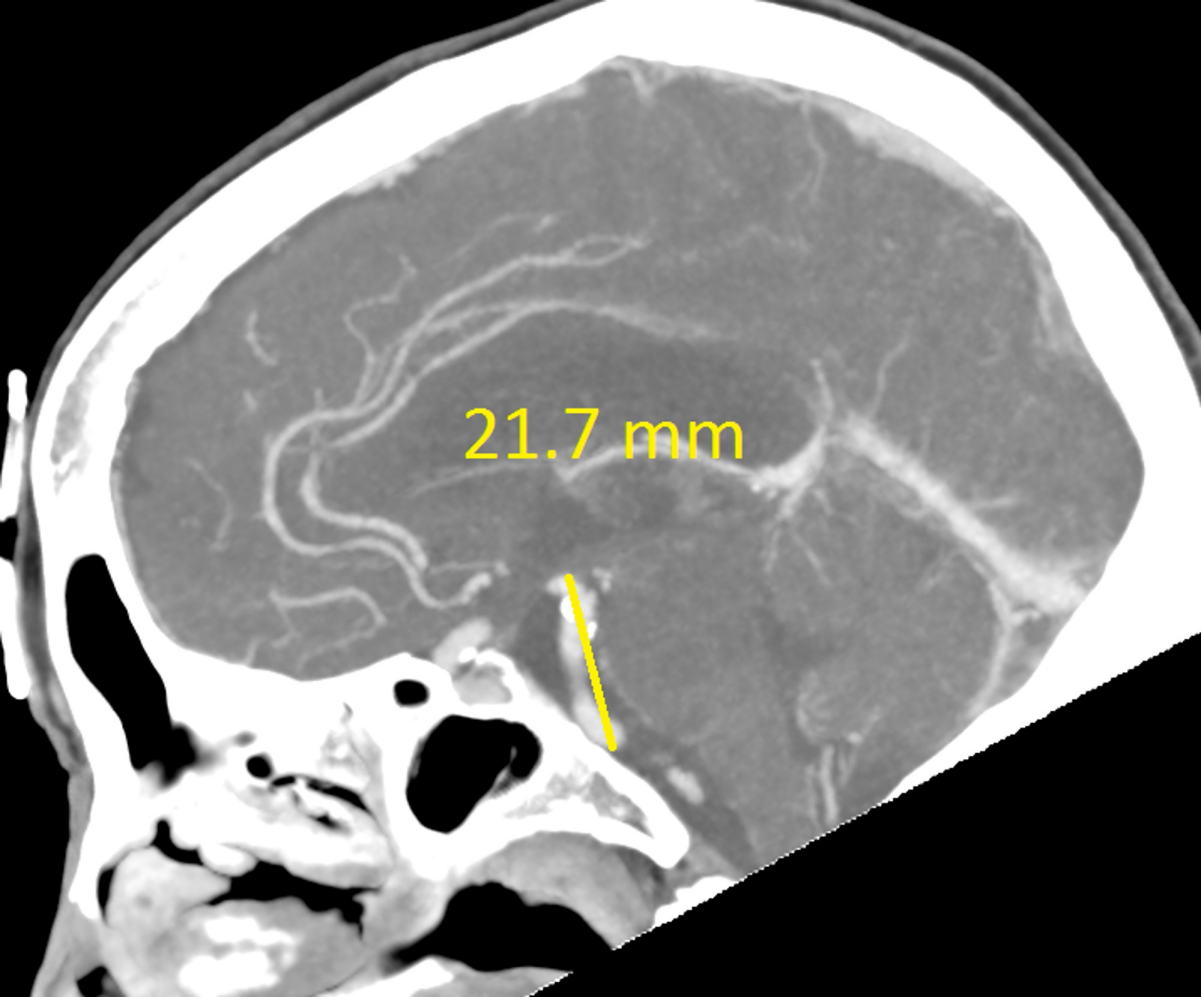
Basilar artery length (part one)

**Figure 4:**
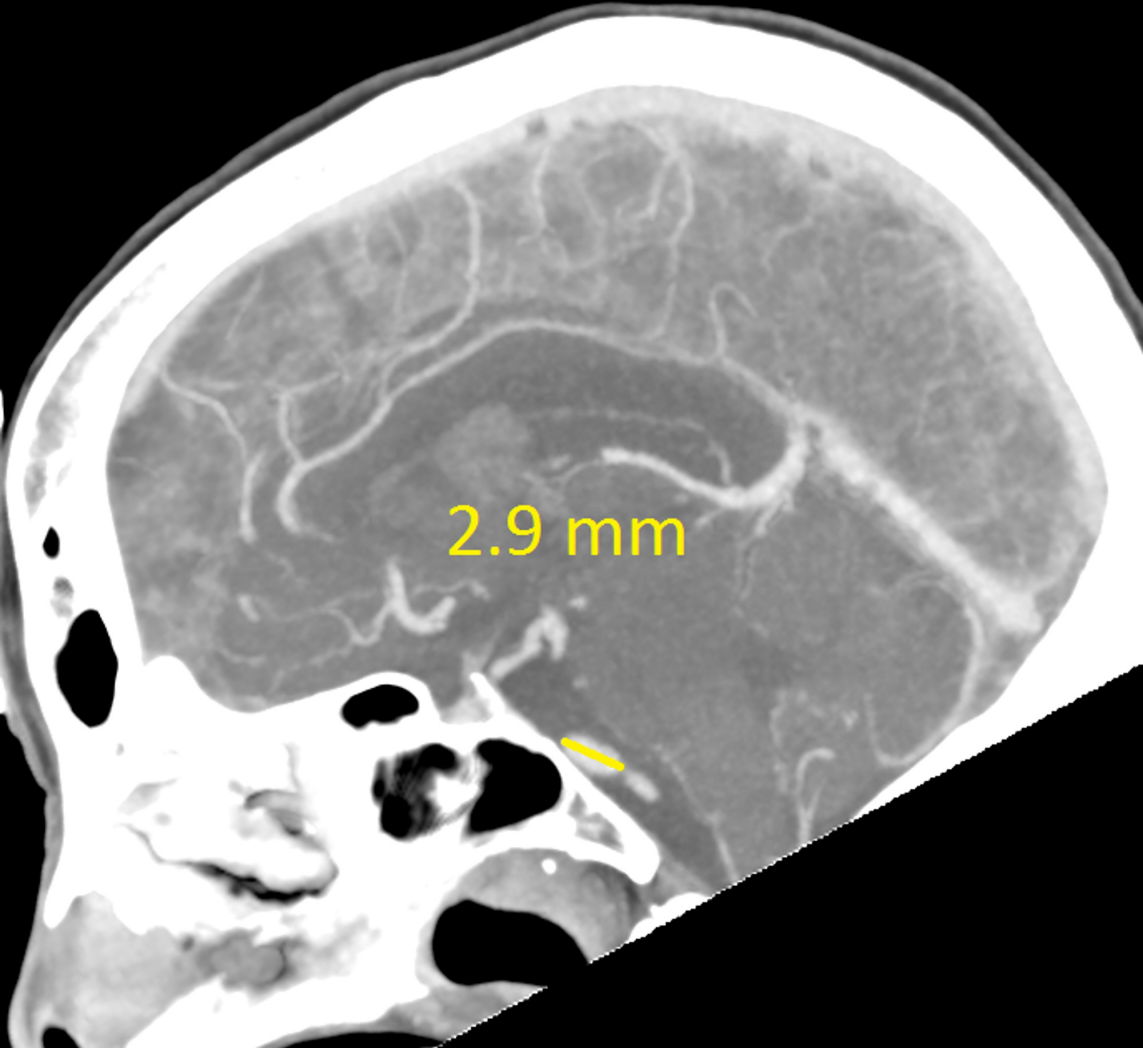
Basilar artery length (part two)

**Figure 5:**
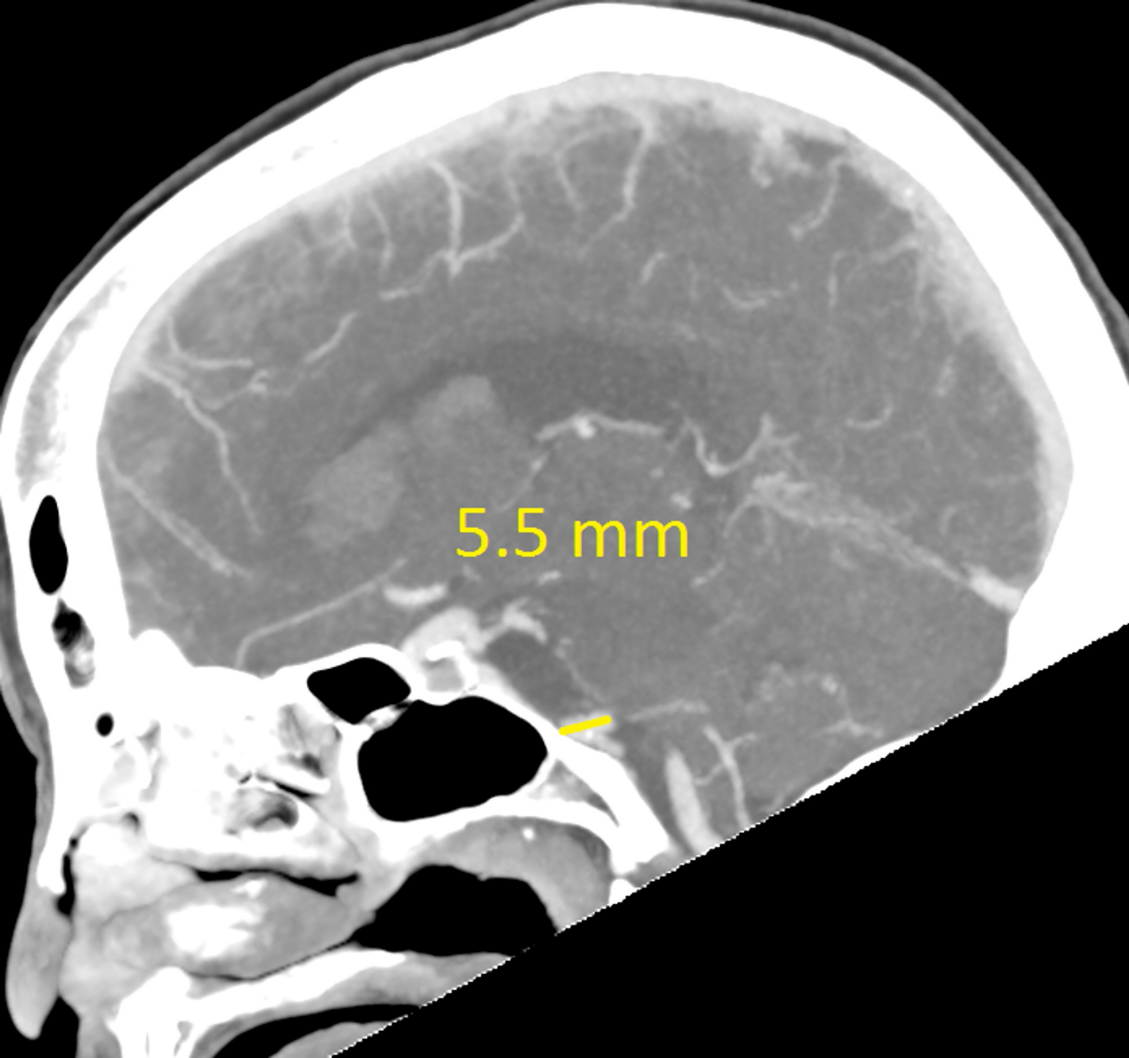
Basilar artery length (part three)

**Figure 6:**
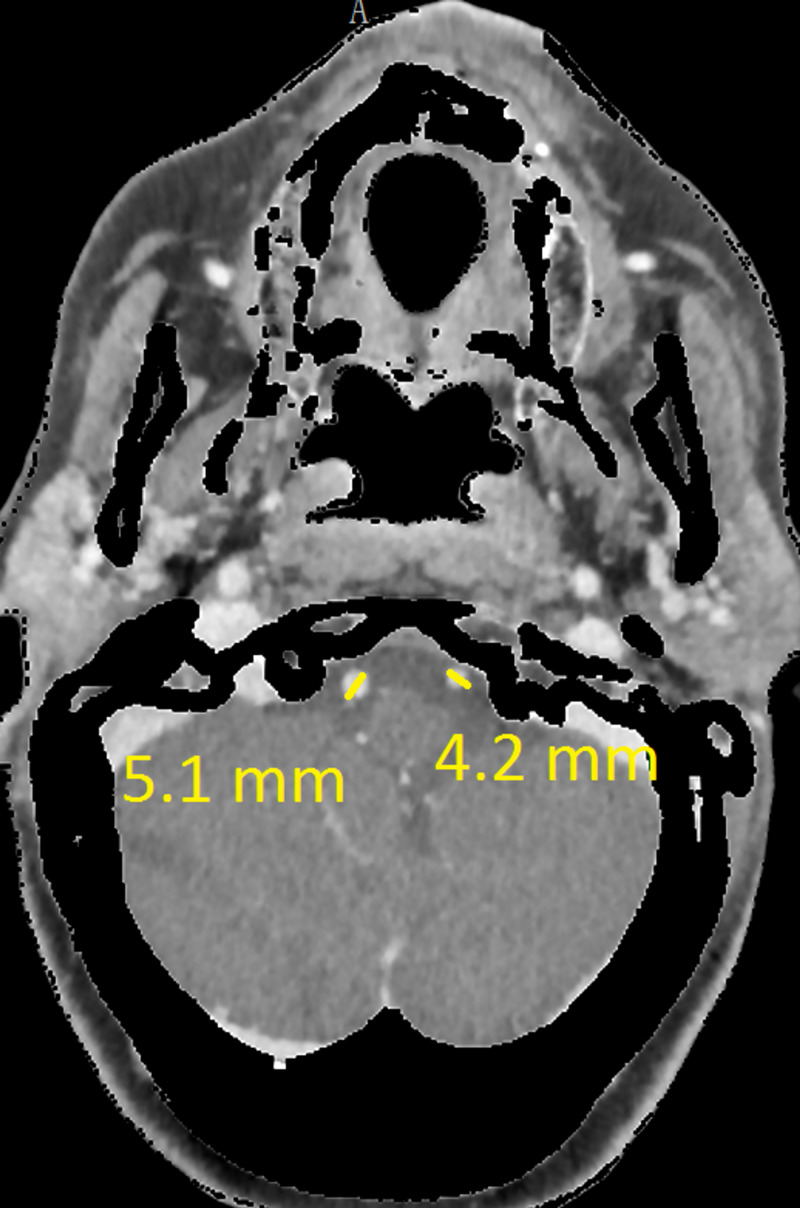
Both vertebral artery diameters

**Figure 7:**
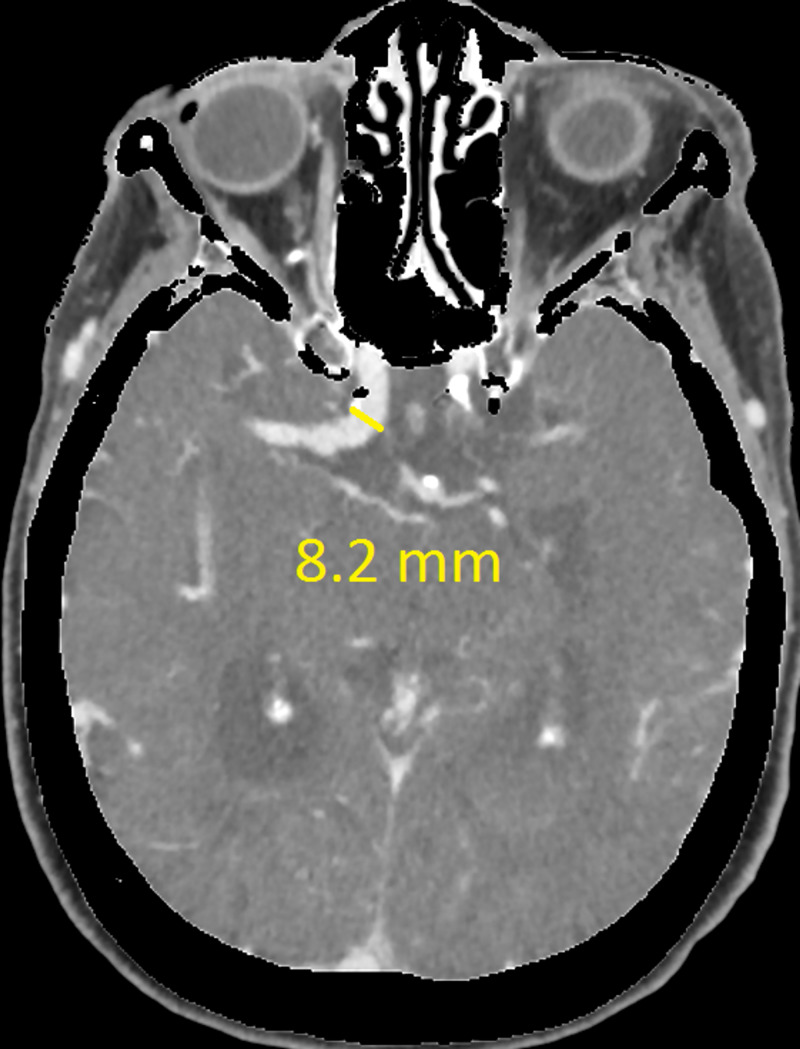
Right supraclinoid internal carotid artery (ICA) diameter

**Figure 8:**
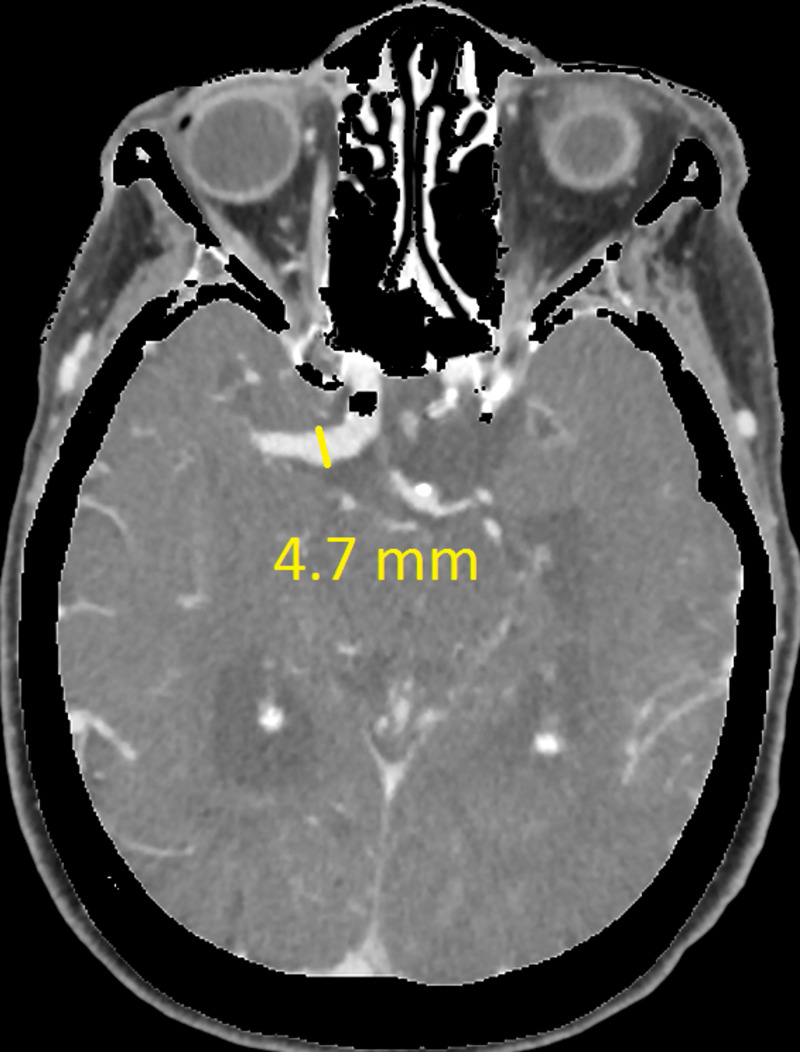
Right middle cerebral artery (MCA) M1 segment diameter

**Figure 9:**
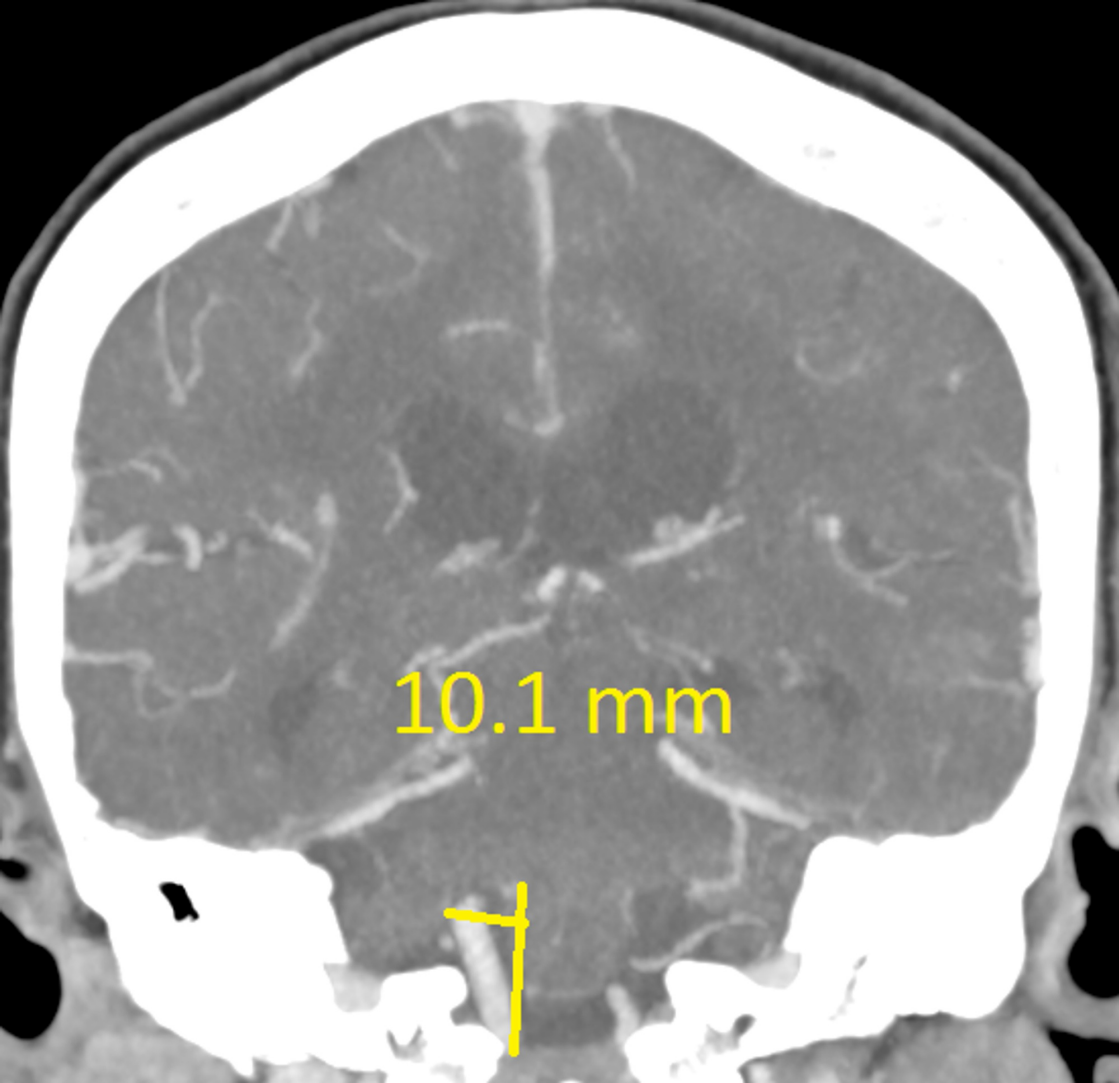
Right vertebral artery deviation

The CT revealed a right temporal intra-cerebral, subarachnoid and intraventricular haemorrhage with enlargement of the Basilar artery and right anterior circulation with marked tortuosity of the Basilar artery. There was good filling of all the vessels with no thrombus formation detected. The Basilar artery diameter measured 7.3mm, was deviated 12.0mm from a perpendicular line connecting the origin of the Basilar artery and its termination and had a basilar length of 30.5mm. The right supraclinoid internal carotid artery (before bifurcation) measured 8.2mm in diameter and the right middle cerebral artery 4.7mm (Figures 1-9).

The haemorrhage had a small area of oedema surrounding it, with no subfalcine herniation and normal ventricle size. After the scan no further seizures were noted and she improved objectively on the Glasgow Coma Scale. She was not considered for emergency surgical intervention after considering the minimal mass effect, her co-morbidities, possible poor surgical outcome and already improving clinical appearance. Best medical treatment was initiated and close neurological monitoring for any deterioration was advised. Her admission course was complicated by a urinary tract infection, with positive cultures. She eventually returned to her baseline and was to be stepped down to a care facility. No further investigations were done to identify aneurysmal dilation elsewhere. She then suddenly passed away in the hospital, a pulmonary embolus suspected to be the cause. The family did not want a post-mortem, therefore we could not confirm the cause of death. 

## Discussion

Cranial arterial dolichoectasia is a term derived from the Greek language meaning both dilation (ektasis) and elongation (dolikhós) of intracranial arteries. When it comes to intracranial dilation a spectrum exists from normal variations to massive aneurysmal dilation causing serious complications [6]. Fusiform aneurysms are a form of non-sacular aneurysm resulting in circumferential ballooning of a vascular segment and this description overlaps with dolichoectasia [7].

The terms dolichoectasia (DE) and fusiform aneurysm can be used interchangeably to describe dilation and increased tortuosity of vessels according to Brorson et al. [8], but DE is usually used to describe a less pronounced form of arterial dilation. According to Brutto et al., fusiform aneurysms refer to extreme circumferential ballooning of the entire vessel wall for a short segment compared to DE which includes less extreme widening with elongation [7]. In an article written by Baran et al. they diagnosed both DE and fusiform aneurysm in the same patient but as different pathologies [9]. Consensus on the definition and extent of DE is unclear and leads to confusion when trying to describe these lesions. Thus, the recent trend in the study of dolichoectasia focuses on dilatation as the main pathologic feature, and consequently, the term “dilatative arteriopathy” has gained popularity [10].

There are various criteria that can be used to classify anterior and posterior dolichoectasia radiologically. For the vertebrobasilar circulation Smoker et al. [11] defined a Basilar artery (BA) diameter greater than 4.5mm at the level of the mid-pons as ‘ectasia’. In contrast, Ubogo and Zaidat [4] defined ‘elongation’ as a BA length more than 29.5mm or a lateral deviation of more than 10mm perpendicular to a straight line drawn from the BA origin and its bifurcation. Gutierrez et al. [12] in 2014 created a more modern definition as a total cranial volume (TCV)-adjusted arterial diameter 2 SD above the population mean. The Gutierrez et al. method is less reproducible as one requires a population mean and an image analysing package to determine the TCV [12].

Concerning the anterior circulation, there are less clear criteria for the diagnosis of dolichoectasia. Baran et al. [9] described a case of circle of Willis DE where the internal carotid artery (ICA) diameter was 11mm and the middle cerebral artery (MCA) 7mm, but used no criteria as guidelines. Fielies et al. [13] described a case of MCA DE that measured 12.5mm but also did not refer to any criteria. In these cases, the vessels were grossly dilated making visual assessment easier and more accurate. But there are few clear criteria available for borderline cases. This is understandable as posterior circulation DE occurs more commonly than anterior DE which has fewer studies to compare to [14]. Passero and Rossi have suggested diameter cutoffs for the ICA (≥7mm), MCA (≥4mm), and vertebral artery (≥4mm) to indicate ‘ectasia’ [15]. 

As mentioned, the posterior circulation is more commonly affected and having both circulations involved is a rare phenomenon [16]. A study done by Brinjikji et al. [3] using data from over 10 years concluded that diffuse intracranial DE is infrequent and carries a worse prognosis. ‘Diffuse intracranial DE’ described adult patients with fusiform aneurysmal dilation of entire vascular segments (supraclinoid ICA, BA, M1 segment of the MCA) that involved two or more intracranial vascular beds (vertebrobasilar system, left anterior circulation or right anterior circulation).

If using the above criteria, the patient in this report has both anterior and posterior circulation DE. For the vertebrobasilar circulation; basilar artery diameter of 7.3mm, lateral deviation of 12.0mm, basilar length of 30.5mm. Anterior circulation; internal carotid artery diameter of 8.2mm, middle cerebral artery diameter of 4.7mm. Diffuse intracranial DE according to Brinjikji et al.; basilar artery segment larger than 6.0mm , M1 segment of the middle cerebral artery larger than 5.0mm, supraclinoid segment of ICA larger than 8.0mm

This is important to recognize - as diffuse intracranial DE (ICDE) is considered a distinct vascular phenotype from isolated vertebro-basilar DE. This was highlighted by the study done by Brinjikji et al. [3]. They found that the age group was older (mean of 70.9 years compared to 60.4 years) and affected a greater proportion of men (84% vs 75.5%). There was also a higher incidence of smoking in the population group affected, concluding that it affects a majority of male smokers with hypertension.

Not only are the risk factors different but also the natural progression. Patients with diffuse ICDE had a higher chance of having abdominal aortic aneurysms (62.5% vs 14.3%) and visceral aneurysms (25.0% vs 0.00%). These patients also had higher and faster growth rates, worse neurological outcomes, greater mortality rate, and an increased likelihood to die secondary to aneurysmal cause. Finally, they also had a greater chance of aneurysmal rupture and ischaemic stroke. This suggests that diffuse ICDE should be considered a systemic vasculopathy rather than an isolated cerebral vascular pathology [14-16].

## Conclusions

Dolichoectasia involving the anterior and posterior circulation or diffuse ICDE is rare, even more so in females that present with intracerebral haemorrhage. This case report highlights the value of using clear and objective criteria for the diagnosis of diffuse DE as it carries a worse prognosis than isolated vertebrobasilar DE. It is usually associated with other aneurysms and has a greater growth rate than vertebrobasilar DE which is important for work-up, management, and follow-up. Criteria for the diagnosis of DE are not widely agreed on but some criteria are more widely used than others. It is mostly found in older males with hypertension, with some studies showing a male predominance of 84%. The main aim of this case report was to highlight the conflict when it comes to the terminology of ectasia and the different objective criteria available for the diagnosis of DE and its importance; also that diffuse ICDE should be considered a systemic vasculopathy and that an extensive work-up should be done to identify other life threatening aneurysm, i.e. abdominal aortic aneurysms. 
